# *S*-Equol, a Major Isoflavone from Soybean, Inhibits Nitric Oxide Production in Lipopolysaccharide-Stimulated Rat Astrocytes Partially via the GPR30-Mediated Pathway

**DOI:** 10.1155/2018/8496973

**Published:** 2018-03-05

**Authors:** Mitsuaki Moriyama, Ayano Hashimoto, Hideyo Satoh, Kenji Kawabe, Mizue Ogawa, Katsura Takano, Yoichi Nakamura

**Affiliations:** Laboratory of Integrative Physiology in Veterinary Sciences, Osaka Prefecture University, Izumisano, Osaka, Japan

## Abstract

Cumulative evidence indicates that estrogen receptor (ER) agonists attenuate neuroinflammation. Equol, a major isoflavone from soybean, exhibits estrogen-like biological activity, but their effect on inflammatory response has not been well established. Here, we investigated the effect of* S*-equol on nitric oxide (NO) production, well-known inflammatory change in astrocytes stimulated by LPS.* S*-Equol attenuated LPS-induced NO production with a concomitant decrease in expression of inducible NO synthase (iNOS).* S*-Equol did not affect LPS-induced increase in intracellular ROS production. Intracellular ER blocker ICI 182.780 had no effect on* S*-equol-induced decrease in NO production. Addition of G-15, antagonist of G protein-coupled receptor 30 which is nongenomic ER and located on cell surface, partially recovered* S*-equol-induced attenuation of NO production. These findings suggest that attenuation of NO production by* S*-equol may mitigate LPS-induced neuroinflammation in astrocytes.* S*-Equol may exert a glioprotective effect, at least in part, via a nongenomic effect.

## 1. Introduction

Recently the crucial role has been recognized for neuroinflammation which is complicated in the pathogenesis of several neurodegenerative diseases such as Alzheimer's disease (AD), Parkinson's disease (PD), and stroke [1, 2]. In these pathological states, microglia and astrocytes are activated; they produce proinflammatory cytokines, such as tumor necrosis factor-*α* (TNF*α*), interleukin-1*β* (IL-1*β*), and interferon-*γ* (IFN*γ*), as well as nitric oxide (NO) and reactive oxygen species (ROS), resulting in neuronal damage; all these changes contribute to CNS disorder [1–3]. Several lines of evidence indicate that estrogen receptor (ER) agonists attenuate neuroinflammation [4–6]. Systemic injection of estrogen inhibits microglial activation induced by intraventricular injection of lipopolysaccharide (LPS) [7]. An ER agonist also reduces TNF*α* and IL-1*β* secretion in cultured astrocytes following LPS treatment [8]. Reduced estrogen level potentiates *β* amyloid peptide deposition in AD model mice [9]. These results suggest that estrogen can alleviate neuroinflammation due to suppressing microglial and astrocyte activation. ER agonists modulate transcriptional activities* via* nuclear ER signaling [10]. In addition to this “genomic” effects, estrogen also acts “nongenomically” on the receptor which is located on plasma membrane, activating multiple signaling pathways that regulate cellular functions [11]. G protein-coupled receptor 30 (GPR30) acts as plasma membrane receptor and shows biological activities of estrogen [12, 13]. GPR30 expressed in microglia contributes to neuroprotective roles in a model of PD and ischemic stroke [14, 15]. However, the roles of genomic and nongenomic effect of ER on neuroinflammation remain to be fully elucidated.

**Figure 1:**
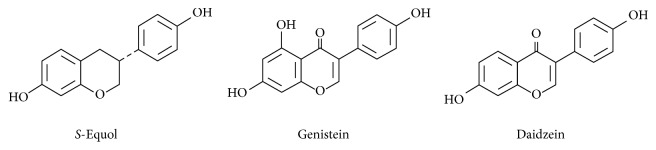
Chemical structures of* S*-equol, genistein, and daidzein.

Isoflavones are natural polyphenolic compounds, which act as phytoestrogens [16] and have several activities such as antioxidant, anti-inflammatory, and antitumor properties [17]. Equol is a major isoflavone compound from soybean. Intestinal bacteria such as* Lactococcus garvieae* metabolize daidzein to* S*-equol [18]. Similar to other isoflavones such as genistein and daidzein, equol exhibits antioxidant property [19]. Because of its similar conformational structure (see Figure 1),* S*-equol shows an affinity of ER and exhibits estrogen-like biological activity [19]. In peripheral, equol inhibits prostate carcinogenesis [20] and also protects and reduces UV-induced skin aging [21]. Such effects are thought to be due to antioxidative effects and induction of apoptosis. In CNS cells, soy isoflavones such as genistein, daidzein, and equol have been reported to be neuroprotective against hypoxia in primary cortical neurons [22]. In glial cells, most of previous work has paid attention to the effect of isoflavones on microglia; genistein [23] and daidzein [24] suppress LPS-induced microglial activation and this is also true for equol [25]. Compared to microglia, there are few reports concerning the effects of isoflavones on astrocytes; genistein suppresses neuroinflammatory changes induced by hemolysate [26] or amyloid *β* [27]. Since the effect of equol on inflammatory responses in astrocytes has not been investigated until now, the role of equol on neuroinflammation is not clear.

LPS, gram-negative bacteria's outer membrane component, directly binds and activates toll-like receptors (TLRs) and its signaling cascades, generating several inflammatory mediators including proinflammatory cytokines such as TNF*α*, IL-1*β*, and IFN*γ*. The LPS-induced changes are thought to mimic those under neurodegenerative diseases [28, 29]. Here, we investigated the effect of* S*-equol on NO production, well-known inflammatory change in astrocytes activated by LPS.

## 2. Materials and Methods

### 2.1. Chemicals and Antibodies

Unless otherwise stated, all chemicals and reagents used in the present study were of analytical grade. Chemicals and antibodies used were as follows: horse serum and Dulbecco's modified Eagle medium (DMEM) from Gibco BRL, Grand Island, NY, USA; LPS from* Escherichia coli* 0127:B8, protease inhibitor cocktail, fetal bovine serum (FBS), trypsin, 2′,7′-dichlorodihydrofluorescein diacetate (H_2_DCFDA), ICI 182.780 (7*α*,17*β*-[9-(4,4,5,5,5-pentafluoropentylsulfinyl)nonyl]estra-1,3,5(10)-triene-3,17-diol), antibodies of anti-*β*-actin and anti-GFAP, and horseradish peroxidase-conjugated goat anti-rabbit IgG (whole molecule) antibody from Sigma Aldrich Corp, St. Louis, MO, USA;* S*-(-)equol from Toronto Research Chemicals, Toronto, ON, Canada; genistein and daidzein from Wako Pure Chemical Co., Osaka, Japan; 2,3-Diaminonaphthalene (DAN), 4′,6-diamidino-2-phenylindole (DAPI), and 3-(4,5-dimethyl-2-thiazolyl)-2,5-diphenyl-tetrazolium bromide (MTT) from Dojindo, Kumamoto, Japan; G-1 (1-[(3a*R*^*∗*^,4*S*^*∗*^,9b*S*^*∗*^)-4-(6-Bromo-1,3-benzodioxol-5-yl)-3a,4,5,9b-tetrahydro-3*H*-cyclopenta[*c*]quinolin-8-yl]-ethanone) from Tocris Bioscience, Bristol, UK; G-15 (3aS,4R,9bR)-4-(6-bromo-1,3-benzodioxol-5-yl)-3a,4,5,9b-tetrahydro-3H-cyclopenta[c]quinolone from Cayman Chemical, Ann Arbor, MI, USA; antibodies of anti-extracellular signal-regulated kinase (ERK)1/2, anti-dual phospho-ERK1/2, anti-p38-mitogen activated protein kinase (MAPK), and anti-phospho-p38-MAPK from Cell Signaling Technology, Inc., Danvers, MA, USA; anti-CD11b antibody from AbD Serotec, Oxford, UK; Immobilon™ Western Chemiluminescent horseradish peroxidase substrate from Millipore Corp., Billerica, MA, USA. iNOS antibody was provided as mentioned previously [30].

### 2.2. Astrocyte Preparation and Cell Culture

**Figure 2:**
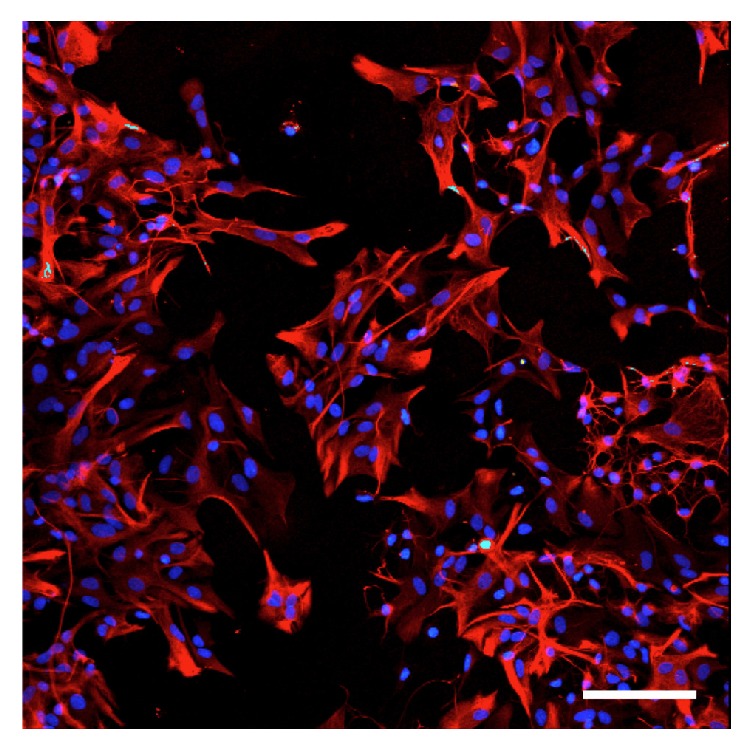
Representative immunohistochemical staining for GFAP (astrocyte, red), CD11b (microglia, green), and DAPI staining (nuclei, blue) in rat primary cortical astrocytes. Scale bar = 20 *μ*m.

This study was approved by the Ethical Committees for Animal Experimentation at Osaka Prefecture University. Rat primary cortical astrocytes were prepared and maintained in DMEM containing 10% FBS, 100 *μ*g/ml streptomycin, and 50 units/ml penicillin in a humidified atmosphere of 95% air and 5% CO_2_ as described previously [31]. For the experiments, astrocytes were replated on day 14 or later into culture dishes or plates (cell density: 4 × 10^5^ cells/ml). In our experiments, there were more than 95% astrocytes as determined by GFAP immunohistochemistry (Figure 2).

### 2.3. NO Measurement

The production of nitrite which is a stable metabolite of NO was measured as previously mentioned [31]. Astrocytes, seeded in 96-well plates, were stimulated with 1 *μ*g/ml LPS for 24 h with or without isoflavones. The concentration of nitrite in cell-free supernatant was determined fluorometrically using DAN reagents with ARVO 1420 Multilabel counter (Wallac, Turuk, Finland; excitation/emission: 355/460 nm).

### 2.4. Cell Viability Assay

The viability of astrocytes was measured using colorimetric MTT assay as mentioned previously [31]. Absorbance at 585 nm was measured using ARVO 1420 Multilabel counter.

### 2.5. Measurement of Intracellular ROS

Intracellular ROS generation was estimated by H_2_DCFDA, the cell-permeable fluorescent dye, as previously described [31]. Briefly, 96-well plated astrocytes were stimulated with 1 *μ*g/ml LPS with or without 50 *μ*M each isoflavones for 3, 6, and 24 h. After that, 5 *μ*M H_2_DCFDA was added to the cells in serum-free medium and incubated for 30 min at 37°C. Dichlorofluorescein (DCF) fluorescent intensity in the cells was measured to estimate ROS generation with ARVO 1420 Multilabel counter, excitation/emission 485/535 nm.

### 2.6. Western Blotting

Cultured astrocytes in 60-mm dishes were stimulated with LPS with or without 50 *μ*M* S*-equol, and subjected to gel electrophoresis followed by immunoblotting as previously described [31].

Immunoblotting was performed using antibodies to iNOS (1 : 10,000), ERK1/2 (1 : 1,000), phosphorylated ERK1/2 (*p*-ERK 1/2; 1 : 1,000), p38-MAPK (1 : 1,000), phosphorylated p38-MAPK (*p*-p38-MAPK; 1 : 1,000), or *β*-actin (1 : 100,000). Protein detection was performed with the aid of enhanced chemiluminescence detection reagents and quantified with LAS-4000 lumino-imaging analyzer (Fujifilm, Tokyo, Japan).

### 2.7. Data Analysis

For estimation of NO and ROS, each group consisting of six culture plates per experiment was studied. Experiments were carried out using five separate seedings of the cells. Data are presented as means ± SEM. The differences between treatments were analyzed by one-way ANOVA followed by the Tukey-Kramer multiple comparison procedure or Student's *t*-test and considered statistically significant when value of *p* < 0.05.

## 3. Results and Discussion

### 3.1. *S*-Equol Attenuated Both Protein Expression of iNOS and NO Production in LPS-Stimulated Astrocytes

It is widely accepted that immune response in the CNS plays critical roles in several neurodegenerative diseases such as AD, PD, and stroke [1, 2]. Under these pathological conditions, inflammatory responses have been employed to stimulate astrocytes and microglia, resulting in activation of pattern recognition receptors including TLRs. Such activation generates inflammatory mediators including proinflammatory cytokines such as TNF*α* and IL-1*β*, free radicals, and NO. LPS directly binds and activates TLR4 and its signaling cascades are thought to mimic those under neurodegenerative diseases [28, 29]. Therefore, stimulating astrocytes with LPS is a useful model to investigate neuroinflammation.

**Figure 3:**
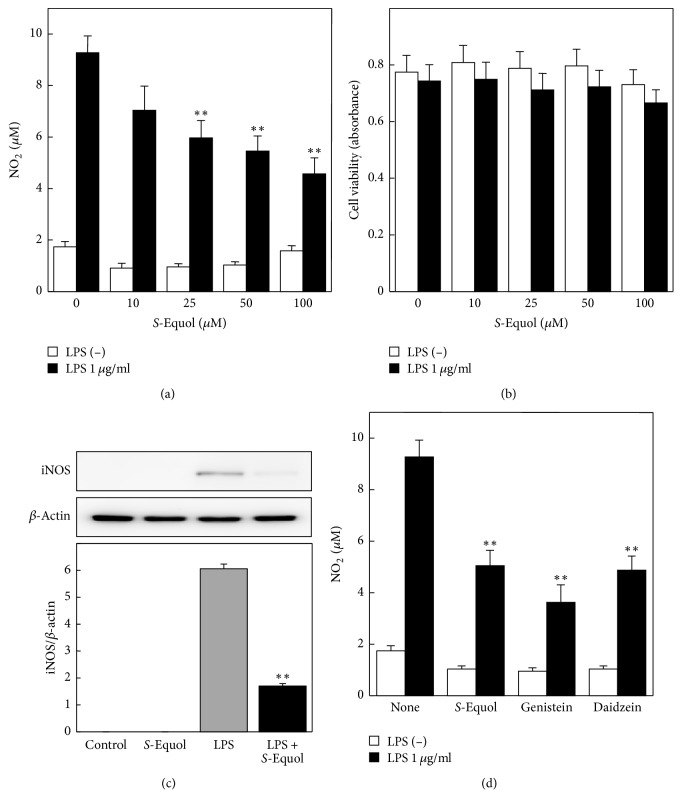
Effect of* S*-equol on LPS-induced NO production, cell viability, and expression of iNOS protein. Cultured astrocytes were stimulated with 1 *μ*g/ml LPS for 24 h in the presence of various concentrations of* S*-equol. The nitrite concentration in the medium was measured fluorometrically (a), and cell viability was evaluated with the MTT assay (b). The cells were treated with 1 *μ*g/ml LPS in the absence or presence of 50 *μ*M* S*-equol for 24 h. The expression of iNOS protein was detected with western blotting (c). Effect of isoflavones (*S*-equol, genistein, and daidzein, each concentration of 50 *μ*M) on LPS-induced NO production (d). Data are the mean ± SEM of 5-6 samples. ^*∗∗*^*p* < 0.01 significantly different from 1 *μ*g/ml LPS.

We first examined whether* S*-equol has an inhibitory effect on LPS-stimulated NO production. Cultured astrocytes were treated with 1 *μ*g/ml LPS in the presence of various concentrations of* S*-equol for 24 h, and NO production in the medium was determined. In the presence of* S*-equol LPS-induced NO production was attenuated in a dose-dependent manner (Figure 3(a)). Significant inhibitory effects were observed with* S*-equol concentration of 25 *μ*M or higher.* S*-Equol, in concentrations up to 100 *μ*M used in this study, did not affect the cell viability (Figure 3(b)).

To explore the mechanism by which* S*-equol inhibits LPS-induced NO production, we next investigated iNOS expression with western blotting. Astrocytes were treated with LPS with or without 50 *μ*M* S*-equol for 24 h; the expressions of iNOS levels were compared. Similar to NO production,* S*-equol significantly inhibited LPS-induced iNOS expression (Figure 3(c)).

We also examined the effect of other isoflavones on NO production. Genistein and daidzein are popular isoflavones found in soybean that also act as phytoestrogens [16, 17]. Similar to* S*-equol, both genistein and daidzein significantly inhibited LPS-induced NO production (Figure 3(d)). This result of genistein confirmed the previous reports that genistein prevents neuroinflammatory changes in astrocytes [26, 27]. These results suggest that these isoflavones reduce neuroinflammatory changes such as NO production in LPS-stimulated cultured astrocytes.

### 3.2. *S*-Equol Had No Effect on LPS-Induced Intracellular ROS Production

**Figure 4:**
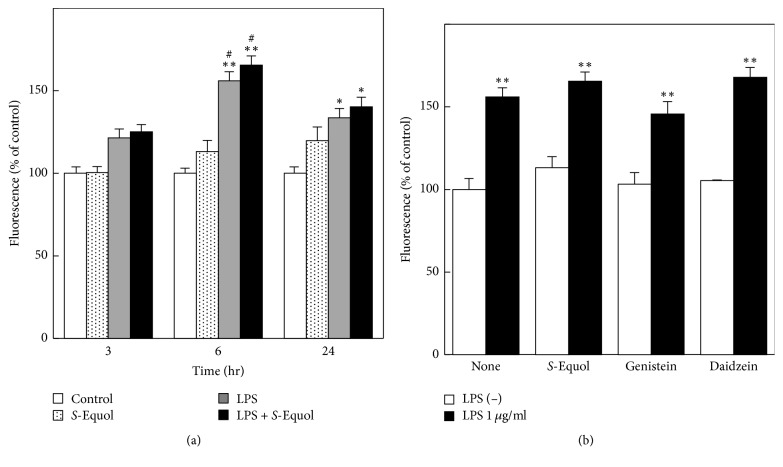
Effect of* S*-equol on LPS-induced ROS production. Cultured astrocytes were treated with LPS (1 *μ*g/ml) in the absence or presence of 50 *μ*M* S*-equol for 3, 6, and 24 h, and the intracellular ROS levels were evaluated using the DCFDA method (a). Effect of isoflavones (*S*-equol, genistein, and daidzein, each concentration of 50 *μ*M) on LPS-induced ROS production at 6 h after LPS treatment (b). Data are the mean ± SEM of 5 samples. ^*∗*^*p* < 0.05; ^*∗∗*^*p* < 0.01 significantly different from the nontreated control. ^#^*p* < 0.05 significantly different from* S*-equol.

The antioxidant property of* S*-equol is well documented in several lines of cells such as macrophages [32] and aortic endothelial cells [33] during inflammation. In addition, treatment of astrocytes with LPS leads to ROS production followed by induction of iNOS expression [34, 35]. Thus, reduced NO production by* S*-equol (Figure 3(a)) might be due to decreased ROS production. To test this, we investigated intracellular ROS generation in* S*-equol-treated astrocytes. Treatment with LPS to astrocytes increased ROS production at 3, 6, and 24 h (Figure 4(a)), as reported previously [31]. Addition of 50 *μ*M* S*-equol failed to decrease LPS-induced ROS production at least within 24 h. Moreover, applying* S*-equol of 100 *μ*M, most effective dose in attenuating NO production in this study (Figure 3(a)), did not mitigate LPS-induced ROS production at 6 h when ROS production was highest in the present study (*S*-equol, 113.2 ± 3.1; LPS, 156.1 ± 5.5;* S*-equol + LPS, 173.7 ± 10.1 in % of control, resp.). Similar results were obtained by using other isoflavones, genistein and daidzein, although each of these isoflavones alone had no significant effect on ROS production (Figure 4(b)). It is unlikely that isoflavones, at least examined in the present study, scavenge ROS and mitigate oxidative stress in astrocytes.

**Figure 5:**
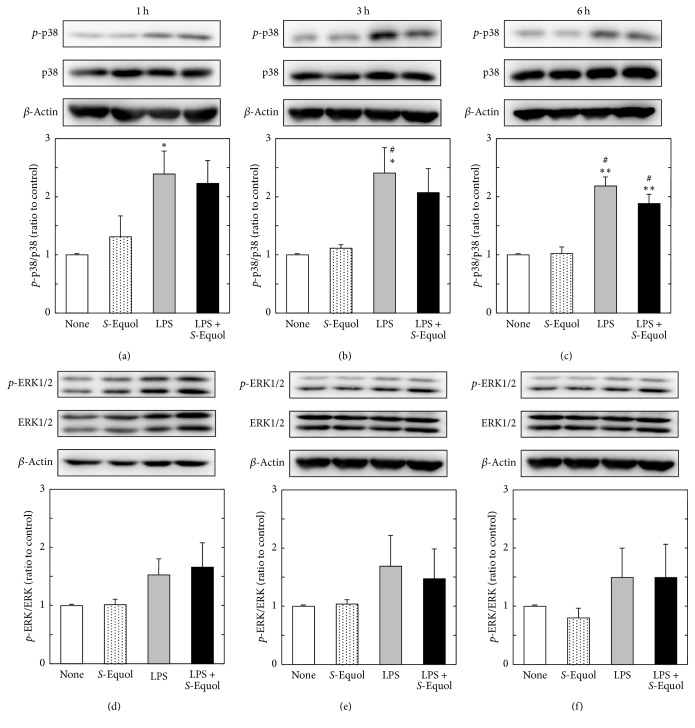
Effect of* S*-equol on LPS-induced MAPK expression. Cultured astrocytes were treated with LPS (1 *μ*g/ml) with or without* S*-equol (50 *μ*M) for 1, 3, and 6 h, and then the expressions of phosphorylated and total p38-MAPK (a–c) and ERK1/2 (d–f) proteins were detected with western blotting. In case of ERK1/2, total density of two bands was analyzed. Data are the mean ± SEM of 4 samples. ^*∗*^*p* < 0.05; ^*∗∗*^*p* < 0.01 significantly different from the nontreated control. ^#^*p* < 0.05 significantly different from* S*-equol.

It is well known that LPS activates MAPKs which mediate intracellular signaling cascades associated with a variety of cellular activities such as cell proliferation, differentiation, survival, and death [36, 37]. To clarify the possible mechanism of* S*-equol-induced suppression of NO production, we checked the effect of* S*-equol on MAPK activation. Astrocytes were stimulated by LPS in the presence or absence of* S*-equol for different time periods (1, 3, and 6 h), and we determined the changes in total and phosphorylated (activated) p38-MAPK and ERK1/2 by western blotting. LPS significantly increased the p38-MAPK phosphorylation at 1, 3, and 6 h. Cotreatment with* S*-equol and LPS had no effect on p38-MAPK activation (Figures 5(a)–5(c)). Also, trend toward increased phosphorylation of ERK1/2 induced by LPS was not affected by the addition of* S*-equol at 1, 3, and 6 h (Figures 5(d)–5(f)). These results suggest that factors other than ROS or MAPKs such as p38-MAPK and ERK may be responsible for the reduction of NO production by* S*-equol. Additional work is necessary to clarify this issue.

### 3.3. *S*-Equol Exerted Its Effect via a Nongenomic Pathway

**Figure 6:**
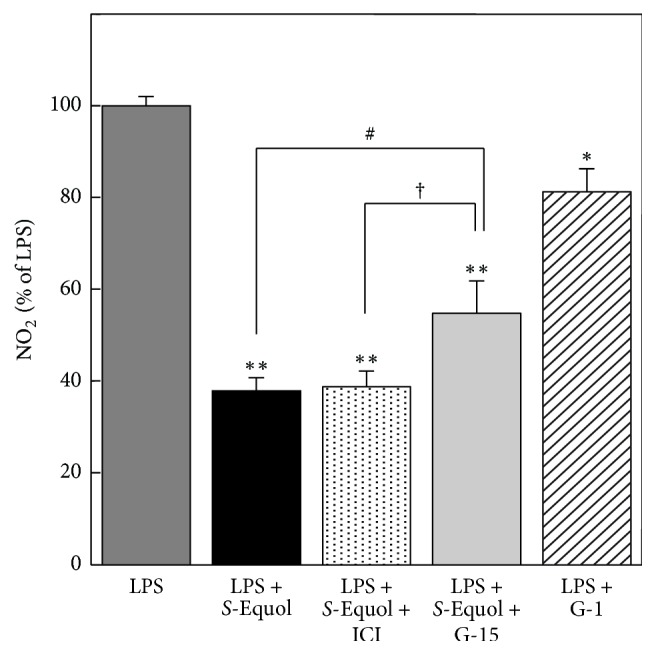
Effect of ER antagonist/agonist on LPS-induced NO production. Cultured astrocytes were treated with LPS (1 *μ*g/ml) and* S*-equol (50 *μ*M) in the presence of ICI 182.780 (an intracellular ER antagonist; 1 *μ*M) or G-15 (a GPR30 antagonist; 1 *μ*M) for 24 h, and NO production was evaluated as described in Figure 3. Some cultures were treated with LPS (1 *μ*g/ml) and G-1 (a GPR30 agonist; 100 nM) for 24 h, and NO production was determined. Data are the mean ± SEM of 5 samples. ^*∗*^*p* < 0.05; ^*∗∗*^*p* < 0.01 significantly different from LPS. ^#^*p* < 0.05 statistical difference between LPS +* S*-equol and LPS +* S*-equol + G-15. ^†^*p* < 0.05 statistical difference between LPS +* S*-equol + ICI and LPS +* S*-equol + G-15.

It is well known that estrogen exerts genomic effects through intracellular nuclear receptor family, ER*α* and ER*β*, that are located in the cytoplasm or on the nuclear membrane [10]. To investigate the molecular mechanism of NO inhibition by* S*-equol in LPS-activated astrocytes, we next examined the effect of ICI 182.780, an intracellular ER antagonist, on NO production. Addition of 1 *μ*M ICI 182.780 had no effect on* S*-equol-induced decrease in NO production (Figure 6).

Recently several lines of evidence reveal that estrogen also acts on the receptor which is located on plasma membrane, activating multiple signaling pathways that regulate cellular function [11–13]. To ascertain whether these nongenomic pathways are responsible for* S*-equol-induced inhibition of NO production, we investigated the effect of agonist/antagonist of GPR30. Addition of 1 *μ*M G-15, antagonist of GPR30, partially recovered* S*-equol-induced attenuation of NO production. Moreover, cotreatment of LPS with GPR30 agonist G-1 (100 nM), instead of* S*-equol, significantly inhibited NO production, although its effect was lesser than that of* S*-equol. These results suggest that* S*-equol attenuates LPS-induced NO production, at least in part, via GPR30-mediated pathway.

The effect of equol on astrocyte function has not been fully investigated previously. In the present study, we revealed, for the first time, that* S*-equol attenuated LPS-induced NO production in astrocytes. Since excessive production of NO aggravates neuronal damage in neurodegenerative diseases [1–3], our results indicate the role of* S*-equol in the attenuation of inflammatory response in CNS. In general, isoflavones are known to be an antioxidant, and, indeed, daidzein has been reported to suppress ROS production in microglia during LPS-induced neuroinflammation [24]. In addition, LPS-induced NO production was inhibited by estrogen which was antagonized in the presence of ICI 182.780 in macrophage [38], indicating that these effects are mediated by classical genomic pathway. In contrast,* S*-equol failed to show antioxidative effect in astrocytes (Figure 4). Therefore, the differences in the effects of* S*-equol on astrocytes and microglia may be attributed to the expression of ER. Moreover, GPR30 antagonist did not completely recover the* S*-equol-induced suppressive effect of NO production, suggesting that other nongenomic pathways such as PI3 kinase-Akt signaling may also be responsible for the effect of* S*-equol [39, 40]. Additional studies are needed to reveal the precise mechanism induced by* S*-equol. Because equol is thought to be easier to pass through BBB than other isoflavones due to its chemical structure [22, 41], our results show new insight into the idea that intake of* S*-equol may be used for managing CNS diseases.

## 4. Conclusions

In summary, our study demonstrates that a soybean isoflavone* S*-equol is a key factor in modulating neuroinflammation induced by the glial activation.* S*-Equol exerts its effect, at least in part, via a nongenomic pathway.

## Abbreviations

AD: Alzheimer's disease
DAPI: 4′,6-Diamidino-2-phenylindole
DCF: Dichlorofluorescein
DMEM: Dulbecco's modified Eagle medium
ER: Estrogen receptor
ERK: Extracellular signal-regulated kinase
FBS: Fetal bovine serum
GPR30: G protein-coupled receptor 30
H_2_DCFDA: 2′,7′-Dichlorodihydrofluorescein diacetate
IFN*γ*: Interferon-*γ*
IL-1*β*: Interleukin-1*β*
iNOS: Inducible nitric oxide synthase
LPS: Lipopolysaccharide
MAPK: Mitogen activated protein kinase
MTT: 3-(4,5-Dimethyl-2-thiazolyl)-2,5-diphenyl-tetrazolium bromide
NO: Nitric oxide
PD: Parkinson's disease
ROS: Reactive oxygen species
TLRs: Toll-like receptors
TNF*α*: Tumor necrosis factor-*α*.
